# A simplified design of a cEEGrid ear-electrode adapter for the OpenBCI biosensing platform

**DOI:** 10.1016/j.ohx.2022.e00357

**Published:** 2022-09-09

**Authors:** Michael T. Knierim, Max Schemmer, Niklas Bauer

**Affiliations:** Institute for Information Systems and Marketing (IISM), Karlsruhe Institute of Technology (KIT), Kaiserstr. 89-93, 76133 Karlsruhe, Germany

**Keywords:** OpenBCI, cEEGrid, Brain-computer-interface, Wearables

## Abstract

We present a simplified design of an ear-centered sensing system built around the OpenBCI Cyton & Daisy biosignal amplifiers and the flex-printed cEEGrid ear-EEG electrodes. This design reduces the number of components that need to be sourced, reduces mechanical artefacts on the recording data through better cable placement, and simplifies the assembly. Besides describing how to replicate and use the system, we highlight promising application scenarios, particularly the observation of large-amplitude activity patterns (e.g., facial muscle activities) and frequency-band neural activity (e.g., alpha and beta band power modulations for mental workload detection). Further, examples for common measurement artefacts and methods for removing them are provided, introducing a prototypical application of adaptive filters to this system. Lastly, as a promising use case, we present findings from a single-user study that highlights the system's capability of detecting jaw clenching events robustly when contrasted with 26 other facial activities. Thereby, the system could, for instance, be used to devise applications that reduce pathological jaw clenching and teeth grinding (bruxism). These findings underline that the system represents a valuable prototyping platform for advancing ear-based electrophysiological sensing systems and a low-cost alternative to current commercial alternatives.

## Graphical abstract

**Figure:**
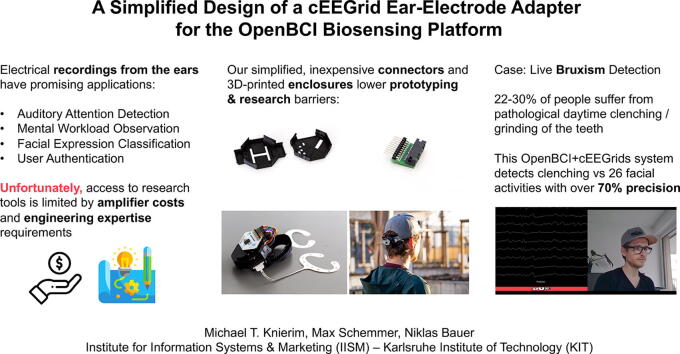

**Specifications table.**

**Table:** 

Hardware name	OpenBCI-cEEGrid Adapter
Subject area	Neuroscience
Hardware type	Electronic engineering and computer science
Closest commercial analog	MBrainTrain Smarting Mobi with cEEGrid Adapter
Open source license	Creative Commons Share-alike 4.0 International
Cost of hardware	Connectors & enclosure: ∼40 USD (main contribution of this work)Amplifier & electrodes: ∼1.500 USD (commercially available)Battery & gel & alcohol wipes: ∼75 USD (commercially available)
OSHWA certification UID	DE000123
Repository link	https://doi.org/10.17605/OSF.IO/ANCFR

## 1 Hardware in context

The OpenBCI-cEEGrid recording system represents an open-source electrophysiological sensing solution that reduces hardware costs and facilitates the development and testing of ear-based wearable prototypes (so-called earables). Ear-based sensing represents an attractive direction for developers of wearable neurotechnologies or human–computer-interaction modalities due to the multitude of detectable signals from within and around the ears and the inconspicuousness with which the necessary hardware can be positioned (ear-pieces, headphones, glasses, or other headwear) [1], [2], [3]. Electrophysiological ear-based sensors have in particular been reported as versatile tools to collect both neural and muscle activity [4], [5], [6], [7]. For neural observation, ear-electroencephalography (ear-EEG) represents a less comprehensive (i.e. without full head coverage), yet more comfortable and applied means of observing brain activity in everyday life. [13,29]. For electromyographic (EMG, i.e. muscle activity) observation, various applications have been proposed that leverage the principle of volume conduction to pick up electrical potentials that propagate through the body to the ear regions. Thereby, while electrodes are placed in less visible recording sites, for example, heart rate activity [6], [7] or facial muscle activity can be observed from afar. This can, for instance, be used to detect different facial expressions of emotions [5], [8].

While such research represents a fascinating and growing development, its progress is impeded by a low degree of accessibility to the required components: (1) cost-effective amplifiers with open data access, (2) ready-to-use ear-(EEG) electrodes, and (3) the integration of both parts into wearable units that can be used in realistic application scenarios. Our presented hardware system aims to lower these research barriers by combining commercially available electrodes (cEEGrids – see [9]) and biosignal amplifiers (OpenBCI Cyton + Daisy – see, e.g., [10]) through low-cost connectors and enclosures with easy assembly. Before this project, OpenBCI ear-based sensing systems had to be individually conceived and assembled with substantial engineering efforts (see, e.g. [11], [12], [13]). Also, the cEEGrid sensors were only usable by designing adapters for expensive EEG amplifiers or purchasing a more expensive commercial analog (e.g., the MBT Smarting Mobi). Thus, the presented project represents a more open, flexible and inexpensive alternative to the current standards. Thereby, we hope to stimulate more research by brain-computer-interface (BCI) and human–computer-interaction (HCI) scholars and enthusiasts interested in developing everyday neurotechnology applications. For the moment, large amplitude and frequency-based observations have been documented. Possible use cases that scholars might be interested in are:

• Multiple hour EEG or EMG recordings in field settings with high comfort and signal quality [7], [14]
• Studying typical EEG phenomena related to auditory stimulation (e.g., detection of directed speaker attention) [7], [15]
• Sleep stage detection [16]
• Mental workload observation [17]
• Detecting facial activities and actions (e.g., as input to control digital applications or to detect facial expressions of emotion) [4], [5]

Altogether, with the exception of sleep stage detection (where additional modifications to the presented system would be required), scholars can readily employ the OpenBCI-cEEGrid system for these various interesting applications in laboratory and field settings. To contribute another application and demonstrate the system’s flexibility, we present new evidence from a use case of particular interest, the observation of jaw clenching events. Repetitive jaw clenching represents a facial muscle activity pattern occurring in daytime bruxism. This condition is associated with multiple physiological and psychological health issues, including fractures, erosion of the teeth, headaches, stress, and anxiety [18], [19]. Results from a machine learning classification analysis highlight that the presented system can identify clench events with a precision of over 70 %, when contrasted with 26 other facial events. We demonstrate this flexibility for application development by providing a biofeedback application with the article that integrates this classifier into an application with real-time predictions (see Chapter 7 for more details).

## 2 Hardware description

To summarize the characteristics of the recording system, it can be said that this simplified OpenBCI-cEEGrid design represents a flexible sensing solution with inconspicuous electrode placement and a platform for the advancement of ear-centered sensing systems due to the open-source nature of the hardware and software. The system contrasts two major options in the field. On the one hand, our solution presents an easy to assemble, multi-electrode sensing system that allows scholars interested in developing earables to utilize available components for easy prototype development. Comparably, previous works that use the OpenBCI amplifiers with ear-electrodes (e.g. for user authentication [11] or attention monitoring [12], [13]) have relied on substantial engineering expertise and personalization to create foam- or plastics-based, customized earbuds (from individual ear shape molds).

On the other hand, our solution represents a comparably low-cost and flexible alternative for scholars interested in conducting ear-EEG research. As previous works (see, e.g. [7], [9]) have primarily relied on using the cEEGrid electrodes with expensive clinical-grade amplifiers (with the most often used, closest commercial analog costing around 8.000 USD – thus at around four times the cost), access to these sensors has been limited to larger budgets. Also, customization options and application development options have been limited (with a notable exception see [20]). Therefore, the utilization of the OpenBCI amplifiers represents a useful alternative due to its open-source nature and availability of hardware and software (see [6]). The software options include a standalone open-source data collection software (OpenBCI GUI) and various application programming interfaces (APIs) to connect the software, e.g. using the established LabStreamingLayer (LSL – https://github.com/sccn/labstreaminglayer – last accessed: August 12th, 2022) protocol which enables precise synchronization of recording timestamps. To compare the OpenBCI platform with commercial analogs, we have comprised the following overview:

• The OpenBCI Cyton/Daisy amplifier provides good signal quality (documented in amplifier comparison studies – see, e.g. [21], [22]) with low input-referred noise (∼1µVpp) considered to be comparable, albeit slightly higher than in commercial analogs [22].
• Collection of up to 18 channels of electrophysiological data – this means fewer channels than in commercial analogs. However, the selection of reference electrode positions is facilitated.
• Recording electrophysiological activity at moderately high sampling rates of up to 250 Hz (on SD card, 125 Hz via live stream) which are, however, lower than other EEG amplifiers which offer 500 Hz or higher sampling rates.
• Long data collection periods, both due to the ability of recording to an SD card and due to the ability to record for an entire day (with a low power consumption of 5mW / channel, a 1000mAh LiPo battery can last for more than 16 h of continuous recordings – which is not possible with the currently closest commercial analog [6], [22].
• Open-source hardware and software allow for further customization and development of software prototypes (which is not as easy with commercial analogs). At the same time, the user-friendliness is slightly reduced compared to commercial analogs as some of the components need to be self-assembled.
• Integration of the system into an active neurotechnology community which facilitates prototype development.

To enable the integration of the electrode and amplifier components discussed above, this article makes two main hardware contributions. First, a connector has been revised to link the cEEGrid ear-electrodes with an open-source biosignal amplifier (OpenBCI Cyton + Daisy). Second, a 3D-printed enclosure has been revised to integrate all required components into a cohesive and sturdy wearable unit.

The adapter is comprised of a printed circuit board (PCB) with a mini edge card socket (that receives the flex-printed cEEGrid electrode) and a set of pin headers to connect jumper wires that are typically used with the OpenBCI amplifier. This design allows for the flexible selection of electrodes on the cEEGrids that can be used for data collection. As the cEEGrids feature ten electrodes (per ear), and the OpenBCI Cyton + Daisy boards can only record 18 channels (including reference and ground), a choice must be made on which electrodes to use. As this decision can influence the phenomenon of interest that is recorded, it is important to have an easy means of re-routing the electrodes included in the recording. In comparison to commercial analogs, this adapter brings a central advantage that the reference and ground electrodes can easily be placed on a particular cEEGrid electrode.

The enclosure is an adaptation of the original Cyton + Daisy board enclosure. As this enclosure is typically used with other OpenBCI components (e.g. the 3D-printed Ultracortex MK IV EEG headset), researchers might want to be able to use this enclosure for multiple purposes. Therefore, most of the design was purposefully kept intact, including the Ultracortex mounting holes on the bottom. However, several iterations were required (see [6] for an earlier version of the system) to identify a useful design for including the cEEGrid connectors. A sturdy slot at the bottom left and right edge was designed to hold the connector PCBs in place. The presented solution does not require any additional tools to mount these connectors. The top of the enclosure completes the solution by holding the connectors in place horizontally so that they do not shift when the cEEGrids are plucked in. To ensure enough space for a battery and the cables below the Cyton board, additional vertical space had to be added to the top cover, and some parts had to be cut out to allow all components to fit together. Lastly, a standalone mounting mechanism had to be devised to put the enclosure on different headgear that researchers might want to use in real-world recording situations. Therefore, the mounting clip was cut out from the bottom of the enclosure.

**Fig. 1:**
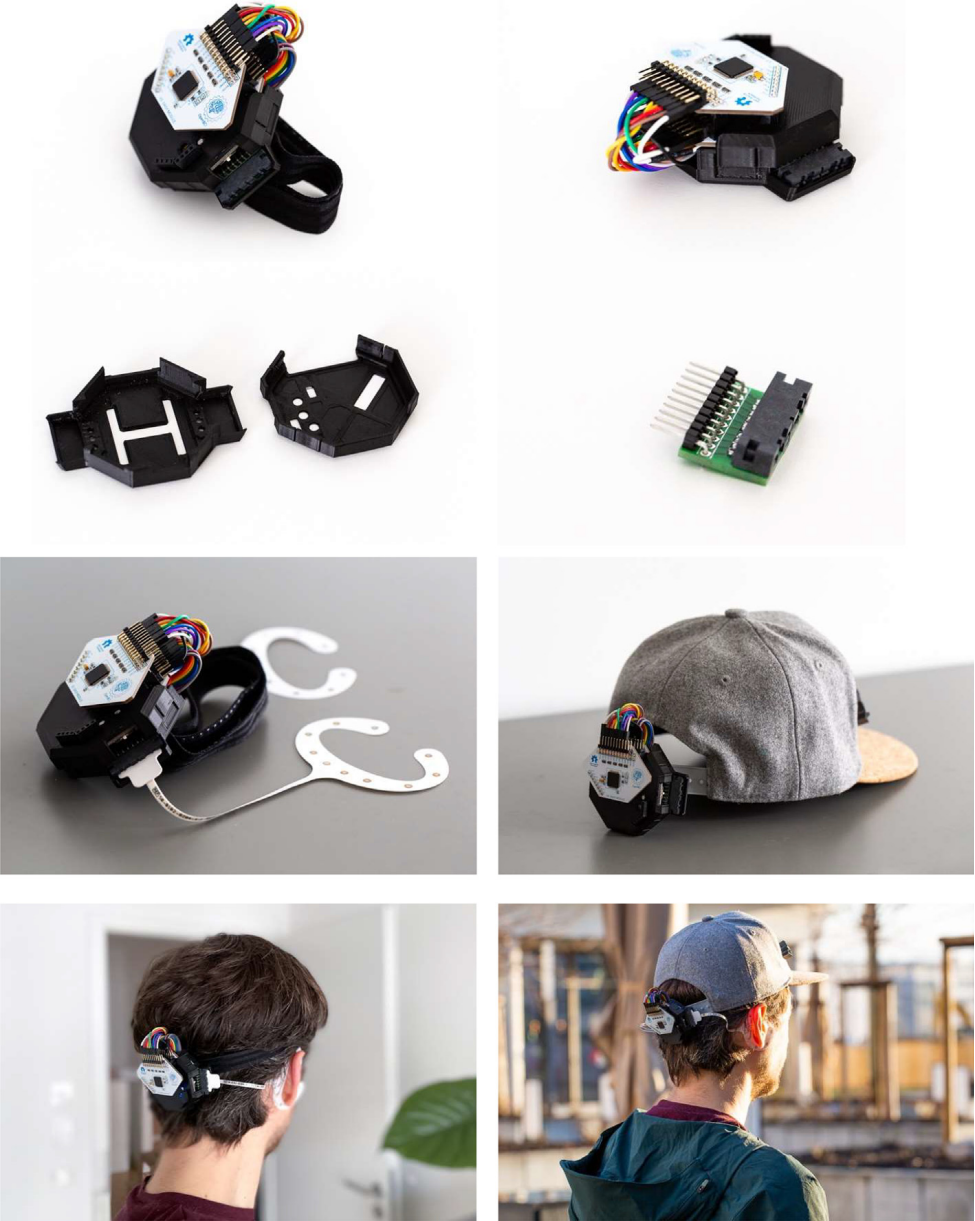
Overview of the components of the OpenBCI-cEEGrid sensing system. The assembled system and the individual components are shown.

In summary, this work presents a refinement over previous developments (see [6]), featuring several advantages over this previous design (see Fig. 1):

• **Better Connector Placement:** reducing the distance to the ears and thereby reducing tension on the electrode cables for some participants with larger head sizes.
• **Better Cable Storage:** By routing the connector cables inside of the enclosure, artefacts from cable contacts are eliminated, and the overall footprint of the system is reduced.
• **Fewer Components:** Through a re-design of the 3D printed components, only two parts need to be printed now (the Cyton + Daisy board cover).
• **Simple Pin Mapping:** Now, a single row pin header is used, which greatly facilitates the process of routing cEEGrid electrodes to the recording channels on the amplifier. Thereby, complex routing schematics (included in previous works) are no longer required.

## 3 Design files summary

The submitted design files comprise the re-designed OpenBCI board enclosures and the printed circuit board that connects the cEEGrids to the amplifier (Table 1). The casings can be adjusted if the specifications of the PCB change (e.g. different size or positioning). The PCB is comprised of a relatively simple mapping pad and via routings to fit a set of pin headers and a mini edge card socket on it. The design files are also made accessible in a permanent digital repository at https://doi.org/10.17605/OSF.IO/ANCFR.

**Table 1:** Design files overview.

**Design file name**	**File type**	**Open source license**	**Location of the file**
Casing Bottom	CAD File & STL File	Creative Commons Share-alike 4.0 International	Available with the article
Casing Top	CAD File & STL File	Creative Commons Share-alike 4.0 International	Available with the article
Connector PCB	PCB Layout	Creative Commons Share-alike 4.0 International	Available with the article

## 4 Bill of materials summary

To complete a single cEEGrid connector, three components need to be sourced. For the entire recording system, two such connectors need to be prepared. The connector PCB can be manufactured by a service like AISLER (https://aisler.net/). An estimate for the production cost is included. The version of the PCB that is shown in the pictures in Fig. 1 was produced with lead free HASL surface finish, two layers, and 1 mm minimal drill diameter. The fourth component listed in Table 2 is the 3D-printed enclosure that houses the entire recording system. An estimate for the printing cost by a manufacturing service (e.g. https://craftcloud3d.com/) is also included in the BOM.

**Table 2:** Bill of materials (connector & enclosure).

**Component**	**Number**	**Cost per unit -currency**	**Total cost -** **currency**	**Source of materials**	**Material type**
Connector Printed Circuit Board (PCB)	2	3.90 EUR	7.80 EUR	Designs available with the article	Other
Mini Edge Card Adapter SAMTEC MB1-120–01-L-S-01-SL-N	2	5.80 USD	11.60 USD	https://www.samtec.com/products/mb1-120-01-l-s-01-sl-n	Metal
Right-Angle Pin Headers (Single Row, 2.54 mm Pitch, 10pins) Molex-0901210130	2	1.45 USD	2.90 USD	https://www.molex.com/molex/products/part-detail/pcb_headers/0901210130	Metal
3D-printed Enclosure (Top & Bottom) – FDM print from a 3D-printing service	1	17.80 USD	17.80 USD	Designs available with the article	Plastic (PLA)

Finally, to complete the recording system, one also needs to purchase the following materials in Table 3.

**Table 3:** Bill of materials (additional components required to complete the system).

**Component**	**Number**	**Cost per unit -currency**	**Total cost -** **currency**	**Source of materials**	**Material type**
OpenBCI Cyton Board + Daisy Shield (Biosignal Acquisition Boards – includes USB-Dongle & Y-Splitter Jumper Cable)	1	1,424.99 USD	1,424.99 USD	https://shop.openbci.com/collections/frontpage/products/cyton-daisy-biosensing-boards-16-channel?variant = 38959256526	Other
Lithium Ion Polymer Battery − 3.7v 500mAh with 2-pin JST-PH connector	1	7.95 USD	7.95 USD	https://www.adafruit.com/product/1578	Other
cEEGrid Electrodes (with adhesive sticker)	2	20.00 USD	40.00 USD	Contact Sales at TMSi (https://www.tmsi.com/contact-us/)	Other
Electrode Gel Abralyt HiCl	1	61.95 EUR	61.95 EUR	https://shop.medcat.nl/Abralyt-hyclate-Dose-van-1-kg?Lng=en	Other
Alcohol Pads	1	3.99 USD	3.99 USD	https://www.walgreens.com/store/c/walgreens-alcohol-prep-pads-isopropyl-alcohol-70/ID=prod6169504-product	Other

## 5 Build instructions

**Fig. 2:**
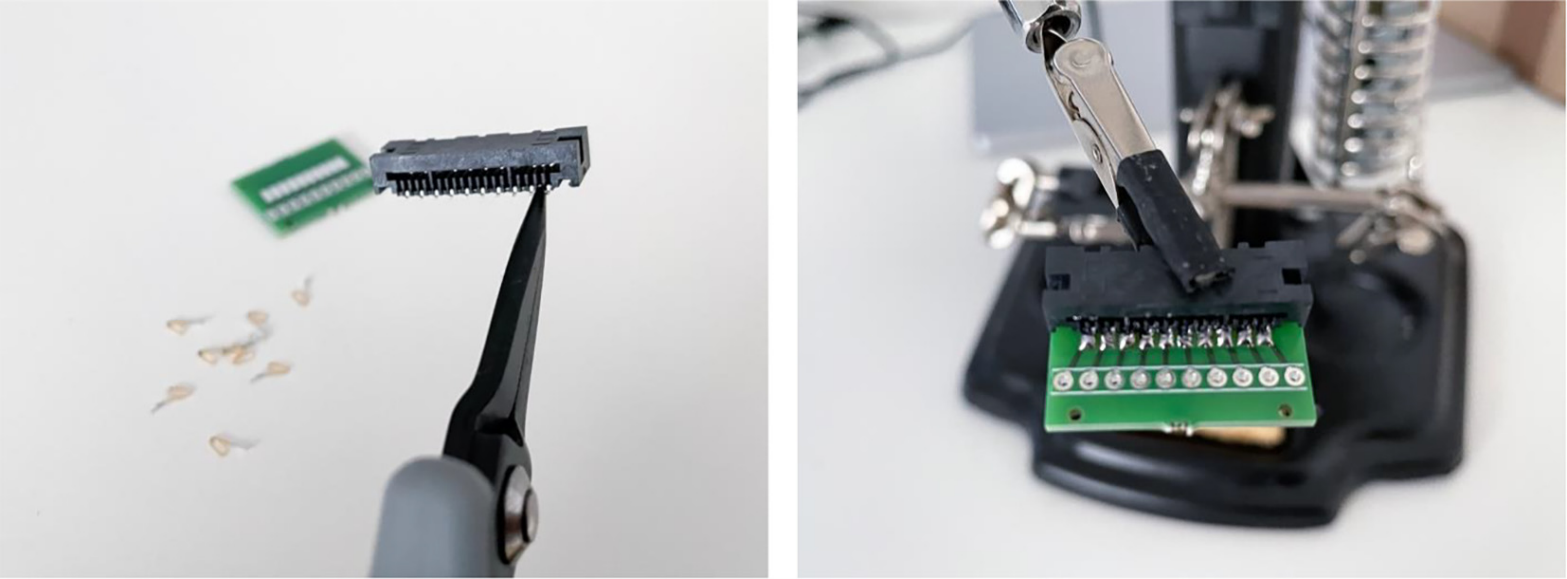
Removing pins from the mini edge card connector (left) and joining the connector components with solder (right).

To assemble the cEEGrid connectors, source the three required components (in Table 2 – i.e. the connector PCB, mini edge card socket, and right-angle pin headers). These parts are joined using a soldering iron. To facilitate the soldering of the mini edge card socket to the PCB and to lower the risk of bridges, it is recommended that every second pin (starting with the first pin) of the card socket is removed before assembling the connector (see Fig. 2). A pair of long snipe nose pliers can be used to easily complete this step. Two connectors need to be assembled for the use of the recording system. Additional instructions for the assembly and for the use of similar cEEGrid adapters are provided by the cEEGrid developers [9] at: https://uol.de/neuropsychologie/howtoconnect (last accessed: August 12th, 2022).

**Fig. 3:**
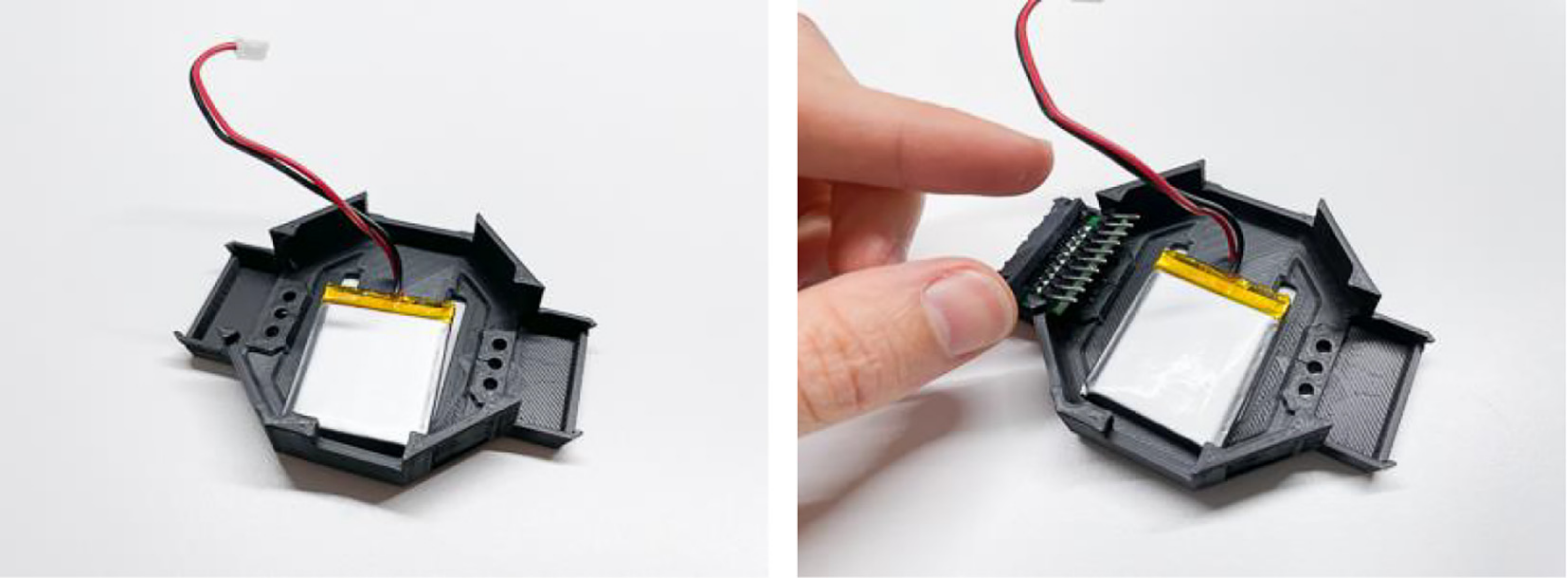
Placing the battery (left) and electrode connectors (right) into the 3D-printed enclosure.

Afterwards, to integrate the connectors with the OpenBCI Cyton + Daisy boards, the connectors need to be fitted into the enclosure, connected to a battery and jumper wires need to be used to map the connector pins to the channels on the amplifier. Initially, the re-designed enclosures (bottom and top) need to be printed. Regular fused deposition modeling (FDM)-printing can be used with a standard 0.4 mm nozzle diameter and 0.2 mm layer height. Rather slow print speeds (e.g. 30–40 mm/s) should be used as the parts have fine details. Once the enclosures are ready, the assembly can start. Place the LiPo battery (here, a 500mAh battery is shown, but a 1000mAh battery also fits) on the bottom of the enclosure. Then, place the connector PCBs in their respective slots. Gently pull on the edges of these slots to allow the PCBs to slide in (see Fig. 3).

**Fig. 4:**
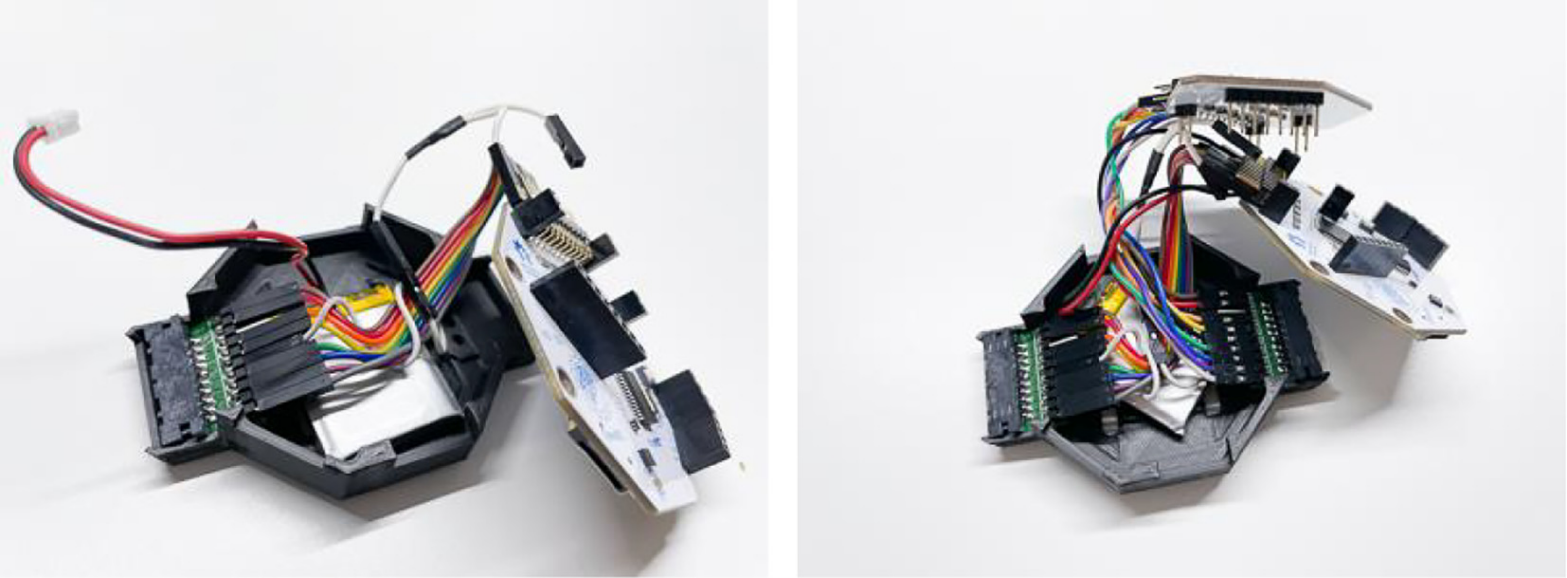
Connecting the connector pins to the amplifier pins via female-female jumper cables. First, connect the left PCB to the Cyton board (left), then connect the right PCB to the Daisy board (right).

Next, the jumper wires can be connected to the Cyton & Daisy boards. Connect the jumper wires to the bottom row of the Cyton/Daisy pins. Use the white y-splitter jumper cable that comes with the purchased Cyton/Daisy package to connect the reference electrode to the first Cyton pin (SRB pin) and the first Daisy pin (SRB pin). In the pictures in Fig. 4, this splitter cable is shown with a connection to the sixth pin (from the top) of the left side electrode connector. Also, connect the designated ground electrode to the penultimate pin (BIAS pin) on the Cyton board. Afterwards, connect the individual connector channels to the amplifier. We recommend routing the left ear to the Cyton pins (channel 1–8 in the recordings) and the right ear to the Daisy pins (channel 9–16). At this stage, it becomes apparent that two connector pins cannot be connected to the Cyton/Daisy board as only 18 pins (incl. reference & ground) are available with this amplifier. For more information on the amplifier and the pin mappings, please refer to the thorough OpenBCI documentation at: https://docs.openbci.com/AddOns/Headwear/MarkIV/ (last accessed: August 12th, 2022).

**Fig. 5:**
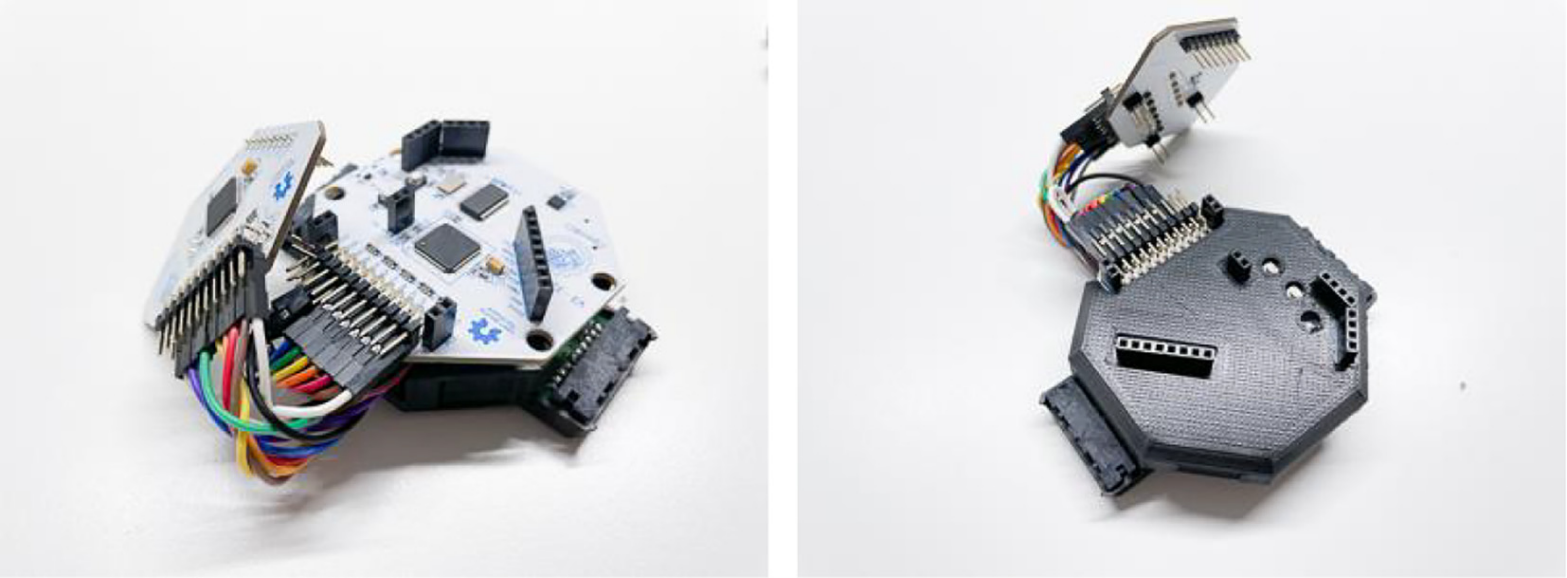
Stacking the assembled components on top of each other to complete the recording unit.

Once this routing is completed, the top of the enclosure and subsequently the Daisy board can be stacked on top of the Cyton board (see Fig. 5). Afterwards, the recording system can be attached to a preferred mounting solution (a headband, cap, hat, VR lenses, or similar), and the cEEGrids can be attached to the card sockets (with the pin surfaces facing down).

## 6 Operation instructions

**Fig. 6:**
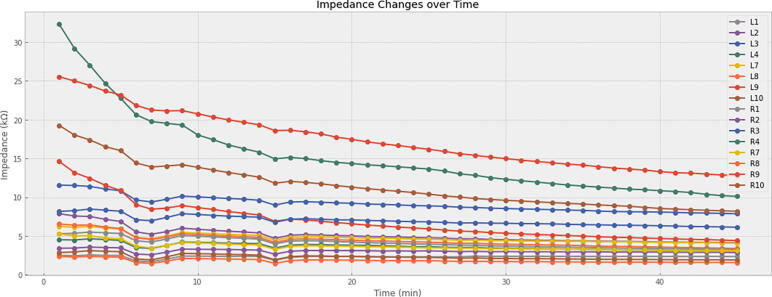
Visualization of impedance changes over time directly after electrode application.

To set up a recording, one needs to initially apply the cEEGrid sensors. Thorough instructions on how to achieve this are provided by the cEEGrid developers [9] at https://uol.de/neuropsychologie/howtouse (last accessed: August 12th, 2022). For completeness, these instructions are adapted here. To ensure high-quality recordings (especially when low-amplitude phenomena like event-related potentials – ERPs – are to be observed), it is important to prepare the skin around the ear (cleaning it from oily residues). Good skin preparation can make a significant difference for achieving high signal-to-noise ratio (SNR). We recommend using pre-packaged alcohol tissues to first clean the area around the ear, followed by a thorough scrub of the skin with an abrasive gel. Especially the latter step has been found to speed up the setup process by achieving lower skin resistances quickly. These resistances also drop after the sensors have been applied for a while – which should be acknowledged when checking the impedances. Therefore, waiting for one to two minutes before performing the impedance check typically helps. To give a more detailed description of these impedance dynamics, Fig. 6 shows the change of impedances over time recorded from a male participant immediately after electrode application (and following the aforementioned skin preparation routine). The impedance traces show that all but one electrode had satisfactory impedances below 30 kΩ right after the electrode application. The 30 kΩ mark has been considered a satisfactory threshold for recording auditory ERPs in previous cEEGrid work [9]. Over time, these impedances settle further. Table 4 shows a selection of impedances for the best, median, and worst electrodes in this recording session. It can be seen that the worst electrode’s impedance drops around ten kΩ after five minutes. The other electrodes’ impedances settle less (0.1 to 0.6 kΩ) in the first five minutes, demonstrating the fast improvement of poor electrodes early on.

**Table 4:** Impedance values over time from selected electrodes.

	**1 min**	**5 min**	**10 min**	**15 min**	**20 min**	**30 min**	**45 min**
**L4 (Worst)**	32.35 kΩ	22.75 kΩ	18.04 kΩ	14.96 kΩ	14.35 kΩ	12.32 kΩ	10.11 kΩ
**L8 (Median)**	6.57 kΩ	5.96 kΩ	5.19 kΩ	4.17 kΩ	4.47 kΩ	3.95 kΩ	3.39 kΩ
**R8 (Best)**	2.36 kΩ	2.27 kΩ	2.08 kΩ	1.47 kΩ	1.89 kΩ	1.71 kΩ	1.57 kΩ

**Fig. 7:**
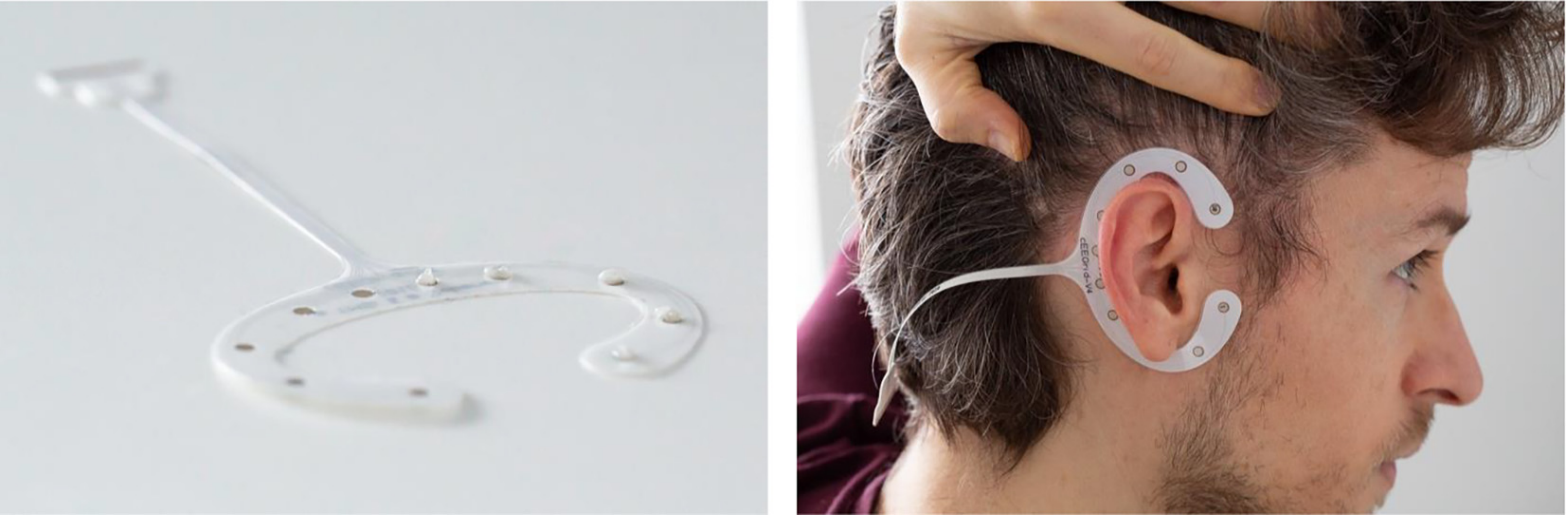
Electrode preparation with gel (left), and placement of electrode on the head (right).

To apply the cEEGrids after skin preparation, peel off the cover of the adhesive sticker and add a drop of electrolyte gel to the cEEGrids (see Fig. 7). We recommend using a syringe (10 ml or 5 ml size) with a blunt needle (e.g. 16G size) to dispense the Abralyt gel. Afterwards, hold back the hair (we have found it helps to pull the hair up towards the tip of the head with one hand – see Fig. 7) to apply the cEEGrid. Take care to not place the electrode too close to the ear base, as the sticker could then put pressure on the skin, which leads to unnecessary discomfort.

**Fig. 8:**
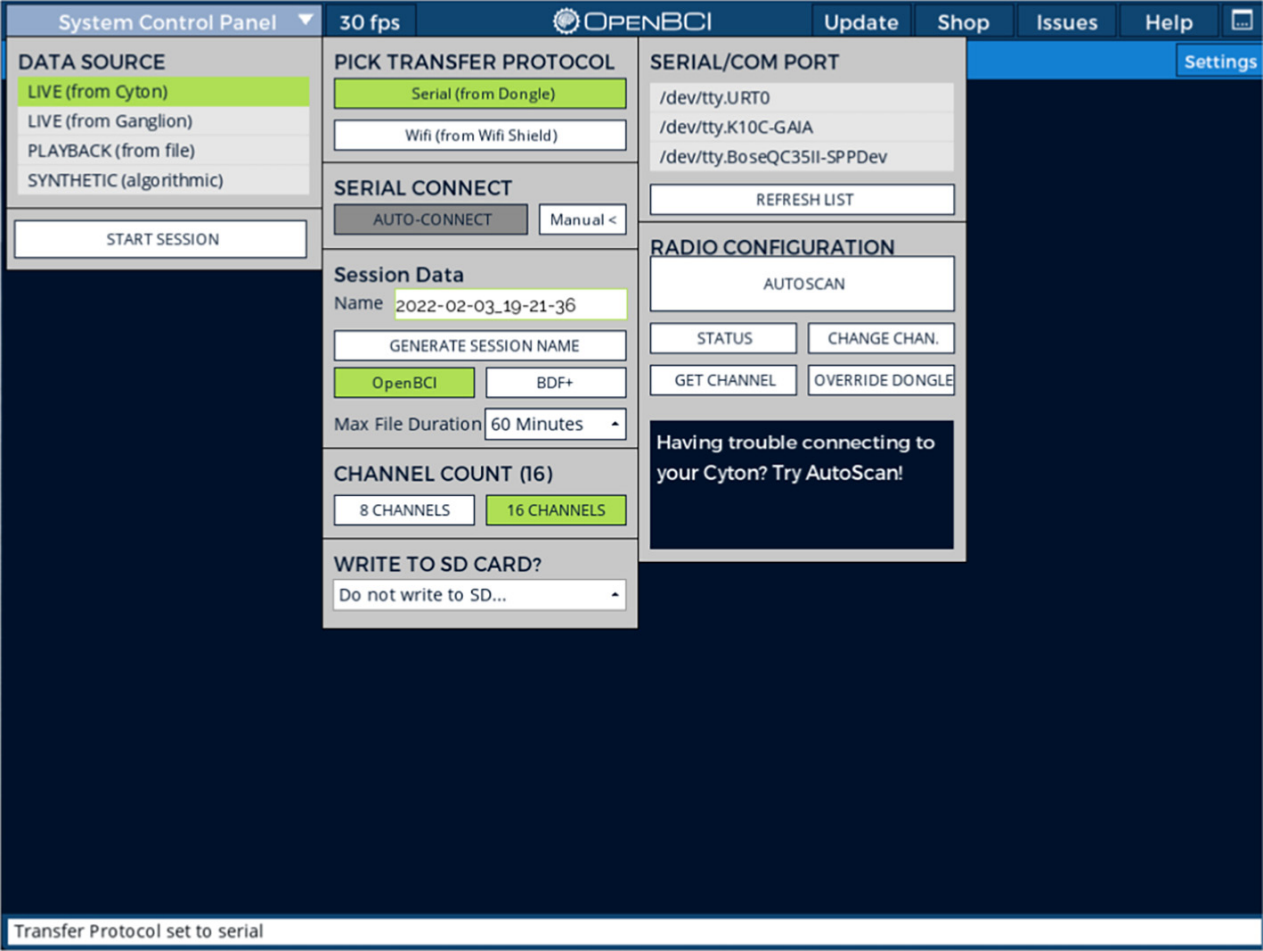
OpenBCI GUI connecting to a Cyton/Daisy amplifier.

To set up a recording, turn on the Cyton board by switching the lever on the right side to the top or bottom position (in the current board version, there is no functional difference between these two positions). The steady blue LED will signal that the board is turned on. To connect the board to a PC, a driver for the Cyton USB dongle and a recording software (here we use OpenBCI GUI) need to be installed. These software solutions are available from OpenBCI (https://openbci.com/downloads - last accessed: August 12th, 2022). Next, connect the USB dongle to a PC and start the recording software. In OpenBCI GUI, start a recording session using the Auto-connect feature (see Fig. 8) or select the COM port with the Cyton dongle manually. Make sure to set the recording to 16 channels (not 8). In this stage, it is also possible to set up an SD card recording. Click on “Start Session” icon in the upper left corner to start a recording.

**Fig. 9:**
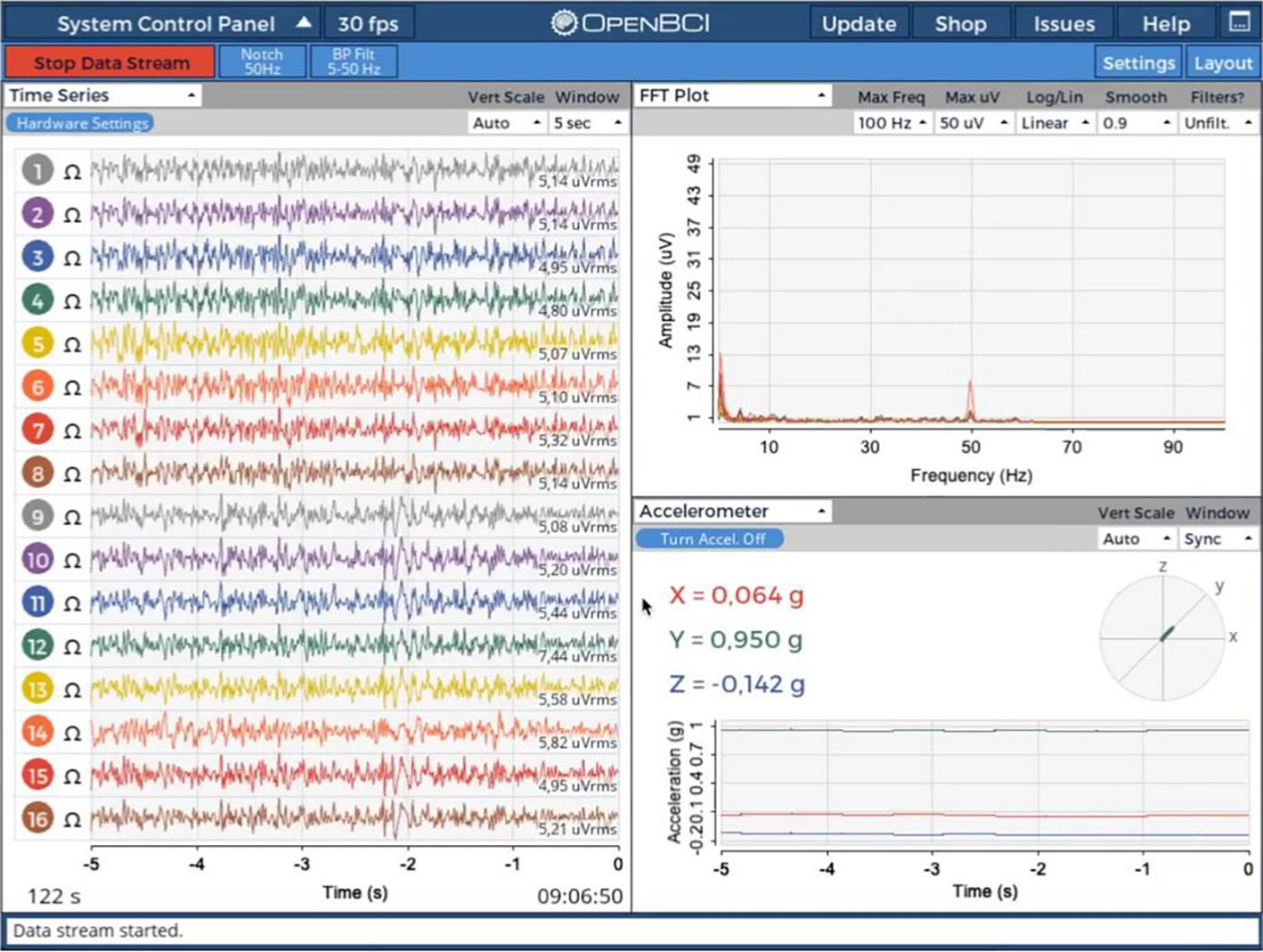
OpenBCI GUI streaming cEEGrid data.

To assess signal quality, multiple functions can be used. First, assess the electrode impedances by toggling the Ω sign next to each channel. As stated above, previous cEEGrid publications have recommended to aim for impedances below 30 kΩ before starting a recording [9]. Second, check the raw signal traces (with a 1–50 Hz bandpass filter) to assess if the amplitudes are plausible and free from excessive environmental contamination (as would be visible in the PSD by large spikes in the local line noise frequency – i.e. 50 Hz or 60 Hz). For example, in the recording demonstration in Fig. 9, Channel 6 shows a high level of 50 Hz line noise and should thus be adjusted (e.g. by adding additional gel to the electrode or by pressing on the electrode firmly to improve the adhesion to the skin). In OpenBCI GUI, the filters are only applied for the display – the data are stored in their raw, unprocessed format. Amplitudes between 5 and 15 µV can be expected when sitting still. Beware that depending on the position of the reference electrode, the amplitudes should be larger on the other head side (e.g. larger on the left side if the reference electrode is placed on the right ear). Similarly, electrodes closer to the reference electrode on the same side typically show smaller amplitudes. Finally, assessing the power distribution can help to ascertain the absence of broadband or high-frequency contamination of the recording (the upper right widget in Fig. 9).

**Fig. 10:**
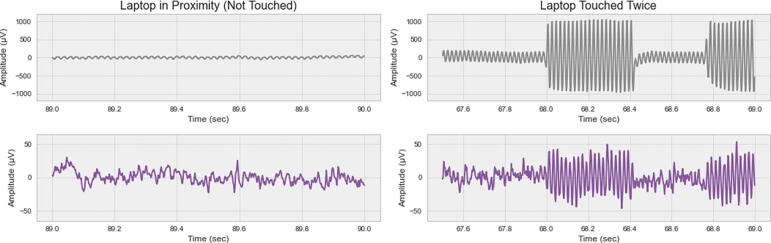
Dynamic line noise contamination patterns in cEEGrid recordings. The top row shows an open electrode (on position L7) that is covered with plastic tape. The bottom row shows a typical recording electrode (position L3).

After having set up a recording, it is still recommended to limit the interaction of the cEEGrid wearer with electrical appliances connected to the power grid (battery-powered appliances are less problematic) and to reduce the amount of movement as both factors can cause large signal artefacts. To give a better impression of such artefacts and to provide instructions on how to handle them, we recorded varying line noise patterns with one participant who wore gelled cEEGrids with low impedances (all < 20 kΩ) and one open cEEGrid electrode. This open electrode (on position L7) was covered with plastic tape to isolate it from electrophysiological signals (i.e., brain & muscle signals). With this setup, the participant repeatedly touched a laptop that was connected to the power grid. Fig. 10 shows the recorded line noise in the open electrode (top row), that is also visible (although with much smaller amplitudes) in a regular recording electrode (bottom row - channel L3 selected). Here, the line noise is present continuously (at low amplitudes) when the laptop was not touched, and it significantly increases when touching the laptop.

During a recording, such line noise bursts could be avoided by using a wireless keyboard and mouse during computer use. In post-processing, conventional line noise removal methods (e.g. notch filters) are often used to remove this sinusoidal artefact pattern. However, notch filtering is often undesirable due to the creation of band-holes, and significant distortion of frequencies around the notch frequency [23]. This means, for instance, that high-frequency EEG bands become unreliable. Such features have been used, for example, in studies on wearable EEG workload estimation in daily-life scenarios where it has been reported that gamma frequency powers possess important discriminatory information [24]. Therefore, for cleaner removal of line noise artefacts, blind source separation methods (such as independent component analysis - ICA) or adaptive regressive filtering approaches (e.g. LMS, RLS, NLMS) are recommended [24]. While the former can help to mitigate line noise, they possess a central limitation when irregularities (non-stationary) patterns emerge, as in the case shown in Fig. 10.

Therefore, given the already available open electrode here and given the multiple additional use cases (discussed below), we decided to show here how an adaptive filter can be used to remove stationary and non-stationary line noise patterns. In principle, adaptive filters use a reference signal (here: the open electrode containing the line noise) to iteratively train a regressive model predicting the recorded signal (here: electrode L3 containing both electrophysiological signals and line noise). Based on this estimated signal, the uncontaminated source signal can be re-constructed. For a more extensive explanation of the method, we refer the reader to [24]. In this report, an adaptive normalized least-mean-squares (NLMS) filter was trained with a window size of 0.25 s and learning rate (mu) of 1. The NLMS filter was chosen as it has been reported to perform well for artefact removal in other EEG data [24].

**Fig. 11:**
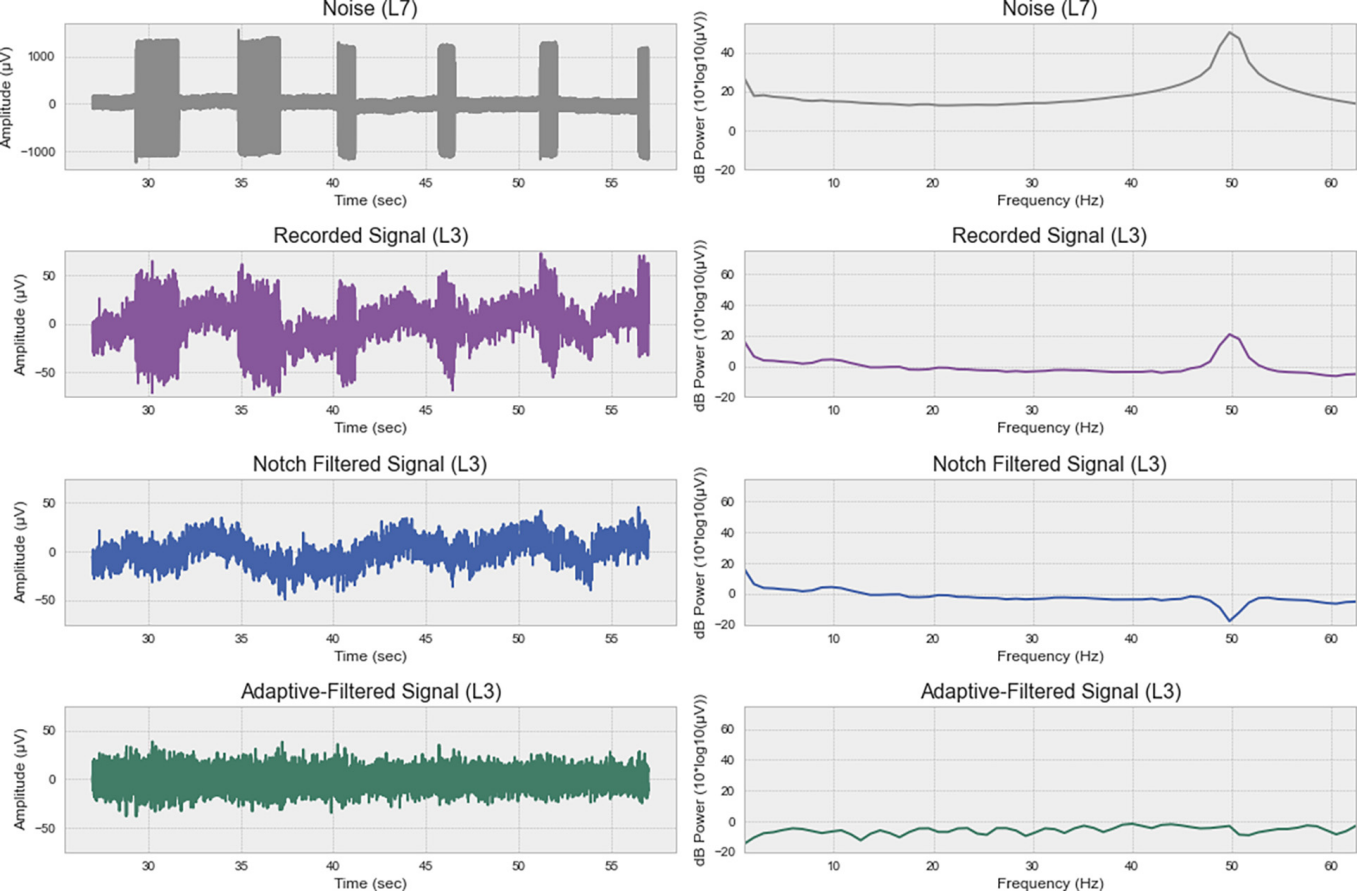
Comparison of line noise removal methods. The first row shows the line noise recorded from an open electrode (position L7). The second line shows the contaminated recording from electrode L3 containing line noise and electrophysiological signals. The third row shows the signal after 50 Hz notch filtering. The fourth row shows the signal after removal of the NLMS-estimated line noise component.

Fig. 11 compares the result of this filter in contrast to a traditional 50 Hz notch finite impulse response (FIR) filter. The power spectral density (PSD) distribution was estimated using Welch’s PSD method with 1 *sec* windows and 0.5 *sec* window overlap. In this figure it can be seen, that the notch filter removes the line noise but leaves a frequency band hole. In contrast, the NLMS filter removes the line noise, without this hole in the 50 Hz region. It can also be seen, that the NLMS filter removes slow trends in the data (baseline wander) that is also recorded in the open electrode. If such an effect is undesired in a particular use case, the adaptive filter could also be applied with a rolling window (e.g. on 2 *sec* windows). Thereby, slow drifts would not be learned by the adaptive filter model.

Interestingly, the use of such an adaptive filter is not only computationally efficient (and thus usable for real time processing applications), it also allows handling movement artefacts in a similar manner. [24] demonstrate that accelerometer signals from a head-worn EEG (which the OpenBCI Cyton also provides for X-,Y- and Z-axes) can also be used to model movement artefacts in the EEG recordings. By employing this technique, the former authors find improved mental workload classification performances during movement (walking and running) and thereby demonstrate how the combination of adaptive filters and wearable EEG can advance ambulatory (i.e. field) studies, for which the cEEGrids are dedicatedly designed for [9]. In summary, to handle electrical and movement artefacts with the OpenBCI-cEEGrid system, we recommend the use of open electrodes and accelerometer signals. An open electrode could, for example, easily be added by covering the electrode opposing the ground electrode with plastic tape. Lastly, for the removal of other non-stationary artefacts (e.g. face muscles), software-based solutions like artefact subspace reconstruction (ASR – see, e.g. [25]) can be used to finalize a comprehensive artefact removal pipeline.

## 7 Validation and application

The OpenBCI-cEEGrid recording system has been demonstrated to replicate well-known and previously documented neural activity findings [6]. Specifically, the former authors documented the blocking of visual input from closing the eyes in occipital brain regions (the so-called Berger effect), and the modulation of higher frequency ranges from changes in mental workload. These observations have previously been established for the cEEGrid electrodes with other amplifiers (e.g., the MBT Smarting Mobi) [7], [9], [17]. In addition, other relatively large amplitude phenomena have been observed with the OpenBCI-cEEGrid combination. Thereby, the distinctive patterns of various facial muscle activities have been found to be captured and differentiated well [4], [5].

**Fig. 12:**
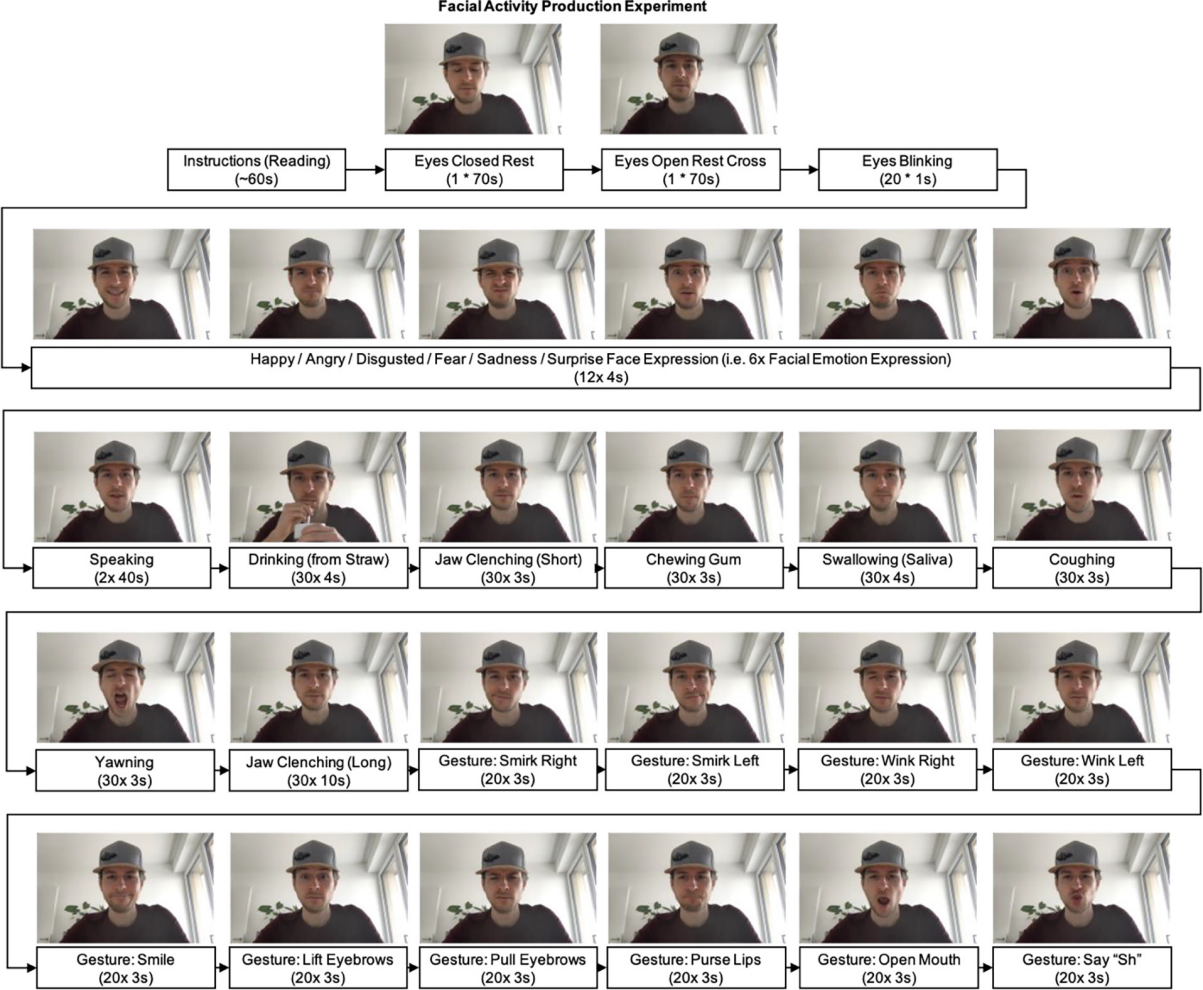
Experiment procedure overview.

In this article, we want to further highlight a promising and relevant use case for this recording system, which is the detection of bruxism events. Bruxism is the repetitive clenching or grinding of the teeth, a condition associated with multiple physiological and psychological health issues, including fractures, erosion of the teeth, headaches, stress, and anxiety [18], [19]. Bruxism occurs during the day and the night (sleep bruxism). Even though prevalence for awake bruxism (AB) is relatively high among adults (22–30 % - compared to sleep bruxism - SB: 1–15 %), its detection and intervention have been researched sparsely [18], [19]. AB detection challenges are that bruxing events need to be differentiated from a variety of other facial activities and that daily recordings need to occur inconspicuously and ergonomically. For this reason, previous work [4] explored whether the cEEGrids can be used with the OpenBCI amplifier as an effective approach to detect bruxing events using machine learning. While this previous work reported promising classification performances (F1-Scores of 0.73 to 0.90 for identifying posed bruxing events vs 13 other posed facial activities), it relied on single-session within-subject classification, which imposes a central limitation towards generalizability and applicability of the detection approach for real-life AB detection. Thus, to further explore the utility of the herein reported sensing system, we extended this previous work by a more complex, multi-session data collection. One participant completed an experiment three times on three separate days in which 28 facial activities were posed (two of which are jaw clenching activities – see Fig. 12 for details). The experimental protocol was highly similar to that reported in [4].

The recorded signals were labeled according to the corresponding experiment phase and trial (with a shift of 350 ms for onset/offset of the facial activity per trial). The short and long jaw clenching phases are thereby considered as bruxism phases. Afterwards, to mimic the process of a live (online) classification system, the data were epoched as non-overlapping 1-second windows. An epoch was considered to contain a bruxism event if ≥ 50 % of the epoch samples were previously labeled as containing a bruxism episode. This processing transforms our multi-class experiment phase into a binary classification problem. The goal is to detect bruxism in the presence of 26 different activities. Due to the experimental setting, our data set contains a data imbalance that needs to be addressed during model training and evaluation (8 percent of the total available data is labeled as bruxism). This data imbalance will also be present in real world applications, as also bruxism-diagnosed patients do not clench their teeth the majority of the day.

Each epoch was then pre-processed using: (1) mean-centering of the channel, (2) band-pass filtering (5–62 Hz IIR), and (3) notch filtering (50 Hz IIR). Next, a range of features was extracted for each channel and epoch: (1) Hjorth Activity, (2) Hjorth Complexity, (3) Hjorth Mobility, (4) Absolute Amplitude Maximum, (5) Absolute Amplitude Sum, (6) Higuchi Fractal Dimension, (7) Petrosian Fractal Dimension. Overall, that lead to a dataset of 9944 epochs (first recording = 3350, second recording = 3315, third recording = 3279) and 16 (channels) * 7 features = 112 features. Two of the recordings were first combined to create a diversified training data set. The third recording was held out as a test data set. To optimize the classifier, fivefold stratified cross-validation was performed using the training data. SMOTE oversampling was used to deal with the imbalanced distribution of classes. All the features were z-standardized and classified with an AdaBoost model.

As we are working with an imbalanced data set (12 percent of the test data set are bruxism events), we use precision and recall to evaluate the performance [26]. Precision measures how many of the predicted bruxism events are true positives. Recall on the other hand measures how many of the bruxism events were detected. The F1-Score is the harmonic mean of precision and recall and provides a single metric for the performance of the classifier. Additionally, we report the Matthews Correlation Coefficient which provides an aggregated measure of all four cases of a confusion matrix [27]. The model was assessed by predicting the unseen test data from the third recording session. This resulted in an F1-Score of 0.73 (Recall: 0.74, Precision: 0.72) for the prediction of bruxing events vs all other 26 classes. With a Matthews Correlation Coefficient of 0.65, we can speak of a positive predictive capability of our model [27].

These results indicate that, while further improvements can be made, the OpenBCI-cEEGrid recording system appears as a viable solution to observe the occurrence of bruxing events. More than 70 percent of bruxism events can be detected. The open-source nature of the hardware and software make the presented system an exciting toolkit for the development of live AB interventions. In the spirit of sharing the content of our research, the recorded data and the code for a simple live bruxism detection system are provided with the article – and in the project repository at https://doi.org/10.17605/OSF.IO/ANCFR.

Altogether, to summarize the characteristics of the recording system, it can be said that this simplified OpenBCI-cEEGrid design represents a versatile sensing solution with inconspicuous electrode placement and a platform for the advancement of ear-centered sensing systems. For the moment, large amplitude and frequency-based observations have been documented to ascertain several application potentials like mental workload or facial activity detection. As the field of ear-centered sensing is further predicted to grow over the coming years [1], [2], the relatively low cost and open-source nature of these components can play a viable role in the conception and testing of ear-centered wearables that have a positive impact on people’s daily lives.

## Ethics statements

The authors of this document confirm that every participant of the experiment mentioned in Chapter 7 has given written consent to the inclusion of material pertaining to themselves.

## CRediT authorship contribution statement

**Michael T. Knierim:** Conceptualization, Software, Investigation, Writing – original draft, Visualization. **Max Schemmer:** Data curation, Writing – review & editing. **Niklas Bauer:** Data curation.

## Declaration of Competing Interest

The authors declare that they have no known competing financial interests or personal relationships that could have appeared to influence the work reported in this paper.
